# Draft Genome Sequence of *Leifsonia rubra* Strain CMS 76R^T^, Isolated from a Cyanobacterial Mat Sample from a Pond in Wright Valley, McMurdo, Antarctica

**DOI:** 10.1128/genomeA.00633-13

**Published:** 2013-08-15

**Authors:** Anil Kumar Pinnaka, Aditya Singh, Sreenivas Ara, Zareena Begum, Gundlapally Satyanaryana Reddy, S. Shivaji

**Affiliations:** CSIR—Institute of Microbial Technology, Chandigarh, Indiaa; CSIR—Centre for Cellular and Molecular Biology, Hyderabad, Andhra Pradesh, Indiab

## Abstract

The 2.7-Mb draft genome sequence of *Leifsonia rubra* strain CMS 76R^T^, isolated from a cyanobacterial mat sample from a pond in Wright Valley, McMurdo, Antarctica, is reported.

## GENOME ANNOUNCEMENT

*Leifsonia rubra* strain CMS 76R^T^ was isolated from a cyanobacterial mat sample collected from a pond in Wright Valley, McMurdo, Antarctica. It is a Gram-positive, nonmotile, curved-rod-shaped bacterium. At the level of the 16S rRNA gene, members of the genus *Leifsonia* exhibited 95 to 96% similarity (1) with *Leifsonia rubra* strain CMS 76R^T^. Whole-genome sequencing of *Leifsonia rubra* strain CMS 76R^T^ was performed using the Roche 454 (FLX Titanium) pyrosequencing platform. The sequencing yielded 127,864,390 bases in 307,943 reads, which is 46× coverage of the genome. Assembly of the raw sequencing data was performed using the GS De Novo Assembler (v 2.8), which yielded a genome of 2,775,399 bp in 207 contigs, with a G+C of 44.31%. All contigs were larger than 500 bp, with the longest contig being 307,711 bp long. Annotation of the assembly data was performed using RAST (Rapid Annotation using Subsystem Technology) (2), tRNA was predicted by tRNAscan-SE (v. 1.23) (3), and rRNA genes were predicted by RNAmmer (v. 1.2) (4).

There are a total of 2,672 protein-coding genes and 51 RNA features in the annotated genome sequence of *Leifsonia rubra* strain CMS 76R^T^. There are 96 genes for membrane transport, including 53 ABC transporters. Twenty-two genes were found to be associated with resistance to antibiotics and toxic compounds. We found 6 genes involved in the synthesis of proteorhodopsin in the annotation of the genome sequence of *Leifsonia rubra* strain CMS 76R^T^; these may be responsible for the yellow coloration of the bacterial colonies. The whole-genome sequencing of the bacterium will help us in understanding the adaptation and evolution of bacteria under adverse climatic condition like the very low temperature conditions of Antarctica.

### Nucleotide sequence accession numbers.

The draft genome sequence of *Leifsonia rubra* strain CMS 76R^T^ has been deposited in DDBJ/EMBL/GenBank under the accession number ATIA00000000. The version described in this paper is version ATIA01000000.
